# Enhanced uptake and transport of PLGA-modified nanoparticles in cervical cancer

**DOI:** 10.1186/s12951-016-0185-x

**Published:** 2016-04-22

**Authors:** Lee B. Sims, Louis T. Curtis, Hermann B. Frieboes, Jill M. Steinbach-Rankins

**Affiliations:** Department of Bioengineering, University of Louisville, 505 S. Hancock, CTRB 623, Louisville, KY 40208 USA; James Graham Brown Cancer Center, University of Louisville, Louisville, KY USA; Department of Pharmacology and Toxicology, University of Louisville, Louisville, KY USA; Department of Microbiology and Immunology, University of Louisville, Louisville, KY USA; Center for Predictive Medicine, University of Louisville, Louisville, KY USA

**Keywords:** Nanoparticles, PLGA, MPG, PEG, Vimentin, Tumor spheroids, 3D cell culture, Cervical cancer, Vaginal epithelium, Cell penetrating peptide

## Abstract

**Background:**

Uncoordinated cellular proliferation and dysregulated angiogenesis in solid tumors are coupled with inadequate tissue, blood, and lymphatic vascularization. Consequently, tumors are often characterized by hypoxic regions with limited access to vascular-borne substances. In particular, systemically administered nanoparticles (NPs) targeting tumor cells and relying on vascular access to reach tumor tissue can suffer from limited therapeutic efficacy due to inhomogeneous intra-tumor distribution and insufficient cellular internalization of NPs. To circumvent these challenges, NP surfaces can be modified to facilitate tumor interstitial transport and cellular uptake.

**Results:**

We create poly(lactic-co-glycolic) acid NPs modified with MPG, polyethylene glycol (PEG), MPG/PEG, and Vimentin (VIM), and evaluate their cellular uptake in 2D (monolayer) cell culture of human cervical carcinoma (HeLa). We compare NP performance by evaluating uptake by non-cancerous vaginal (VK2) cells. We further assess NP interstitial transport in hypo-vascularized lesions by evaluating the effect of the various modifications on NP penetration in 3D cell culture of the HeLa cells. Results show that after 24 h incubation with HeLa cells in monolayer, MPG, MPG/PEG, PEG, and VIM NPs were internalized at 66×, 24×, 30×, and 15× that of unmodified NPs, respectively. In contrast, incubation with VK2 cells in monolayer showed that MPG , MPG/PEG , PEG , and VIM NPs internalized at 6.3×, 4.3×, 12.4×, and 3.0× that of unmodified NPs, respectively. Uptake was significantly enhanced in tumorigenic vs. normal cells, with internalization of MPG NPs by HeLa cells being twice that of PEG NPs by VK2 cells. After 24 h incubation in HeLa 3D cell culture, MPG and MPG/PEGNPs were internalized 2× and 3× compared to PEG and VIM NPs, respectively. Whereas MPG NPs were internalized mostly in the cell culture periphery (1.2×, 1.4×, and 2.7× that of PEG, MPG/PEG, and VIM NPs, respectively), PEG NPs at 250 μm penetrated 2× farther into the tissue culture than MPG NPs. For all NP types, cellular internalization was severely hindered in 3D compared to monolayer.

**Conclusions:**

Although MPG surface modification enhances internalization and uptake in hypo-vascularized cervical tissue culture, coating with PEG reduces this internalization while enhancing penetration. A delivery strategy combining NPs with either modification may balance cellular internalization vs. tissue penetration in hypo-vascularized cervical cancer lesions.

**Electronic supplementary material:**

The online version of this article (doi:10.1186/s12951-016-0185-x) contains supplementary material, which is available to authorized users.

## Background

Cervical cancer is the third most common gynecologic cause of cancer associated with patient fatalities. Approximately 13,000 new cases of invasive cervical cancer are diagnosed yearly, of which 30 % prove fatal. In the US, cervical cancer primarily afflicts women younger than 50; however, in countries without established screening and prevention programs, cervical cancer remains the second most common type of cancer and cause of death among all female cancers [1–3]. Screening tests and vaccines have contributed to a decrease in cases; to date, there are 3 approved vaccines against cervical cancer. These vaccines, Gardasil^®^, Gardasil^®^ 9, and Cervarix^®^, aim at preventing cancers originating from HPV types 16 and 18, which are attributed to ~70 % of cervical cancers [4]. Yet despite this efficacy, the vaccines only protect against a subset of all known HPV strains [4–6]. The inadequacy of vaccination, coupled with the fact that vaccination is not widespread [5, 6], maintains the risk of cervical cancer as a fatal disease.

Relative to the success of prophylactic vaccines, ineffective treatment options exist for established HPV infections and cervical cancer originating from HPV. This is primarily attributed to multidrug resistance and chemotherapeutic side effects. Despite early stage tumor identification and established eradication methods including surgery and radiation, the adverse side effects of these treatment strategies often result in negative gynecologic and obstetric outcomes. In comparison to surgical and radiation challenges, even systemic chemotherapy results in relatively low transport efficiency, resulting in high chemotherapeutic doses needed to target mucosal and epithelial tissue [7–10]. Due to the high systemic toxicity induced by systemic chemotherapy, new treatment strategies are urgently needed.

In addition to these clinical challenges, successful therapeutic agent delivery to the tumor microenvironment requires minimizing agent degradation and excretion, avoidance of immunogenic interactions, adequate penetration and distribution throughout the tumor tissue, cellular uptake and internalization, and sufficient cytotoxicity [11]. United States Food and Drug Administration (FDA)-approved materials may be selected as nano-scale drug carriers that have been proven to be non-inflammatory and non-toxic while enabling the delivery of highly localized concentrations of both hydrophilic and hydrophobic agents [11–13]. In particular, polymer nanoparticle-based drug delivery systems have been evaluated as attractive options for efficacious delivery of agents such as drugs and genes with resulting treatment efficacy [12, 13]. The pharmacokinetics of polymeric systems can be tailored by changing the polymer monomer concentrations to facilitate agent diffusive transport, typically via a combination of burst and sustained release profiles.

Besides enabling the sustained release of encapsulated agents, relative to systemic options, local intravaginal therapy may represent a relatively non-invasive approach to locally treat cervical tumors [7]. Nanoparticles (NP) can be modified to minimize unwanted systemic interactions, prolong bioavailability, promote targeted delivery to the physiologic site of interest, and enhance tumor penetration and cellular uptake [14]. Different types of NP surface modifications can be utilized to elicit these desired behaviors. Stealth coatings such as polyethylene glycol (PEG) can decrease unwanted systemic interactions and neutralize the carrier’s surface charge [15, 16], while targeting ligands can increase the specificity of conjugated carriers to systemically target the desired physiological environment [17]. Cell penetrating peptides, such as MPG, RGD, and TAT, are examples of short ligands that can enhance the cellular internalization of their conjugated carriers [18–29]. In particular, previous studies have shown that NP surface modification with the amphipathic, synthetic peptide MPG derived from HIV gp41 and SV40 [24, 30] displays high cellular binding and internalization achieved via clathrin-mediated endocytotic uptake [31].

Tumor tissue is typically characterized by an overabundance of extracellular matrix as well as poorly vascularized areas, both of which can hinder diffusive transport of NPs [11]. Thus, in addition to evaluating cellular uptake capability, delivery systems would benefit from testing in a three-dimensional (3D) environment which more closely represents hypo-vascularized tumor lesions. For this purpose, 3D cell culture provides a suitable platform [32, 33] that is more controllable than tumors in vivo. Previously, 3D cell culture has been employed to study the behavior of various nano-carriers, such as gold and polymeric NPs. It has been shown both experimentally [34–38] and theoretically [39–49] that NPs require appropriate characteristics and surface modifications to adequately penetrate hypo-vascularized regions for effective therapeutic delivery, even if cellular internalization is optimized.

With the goal to enhance both the transport and internalization of therapeutic agents in hypo-vascularized tumor tissue, in this study we evaluate a variety of poly(lactic-co-glycolic) acid (PLGA) NP formulations for tissue penetration and cellular internalization. The NPs were modified with either a cell penetrating peptide (MPG), a stealth ligand (PEG), a tumor targeting cell penetrating peptide (Vimentin tubulin binding site peptide, VIM) [50, 51], or a hybrid modification with both cell penetrating peptides and stealth ligands (MPG/PEG). These ligands were chosen because they have already been individually well characterized to enhance cell internalization, e.g., as we have previously shown for MPG [31]. We assess how the NP surface modifications affect uptake in cervical tumor cells, and compare the NP performance in normal vaginal cells. We further evaluate the NP transport and internalization in a 3D spheroid model representing hypo-vascularized cervical cancer lesions.

## Results

### Nanoparticle characterization

**Fig. 1:**
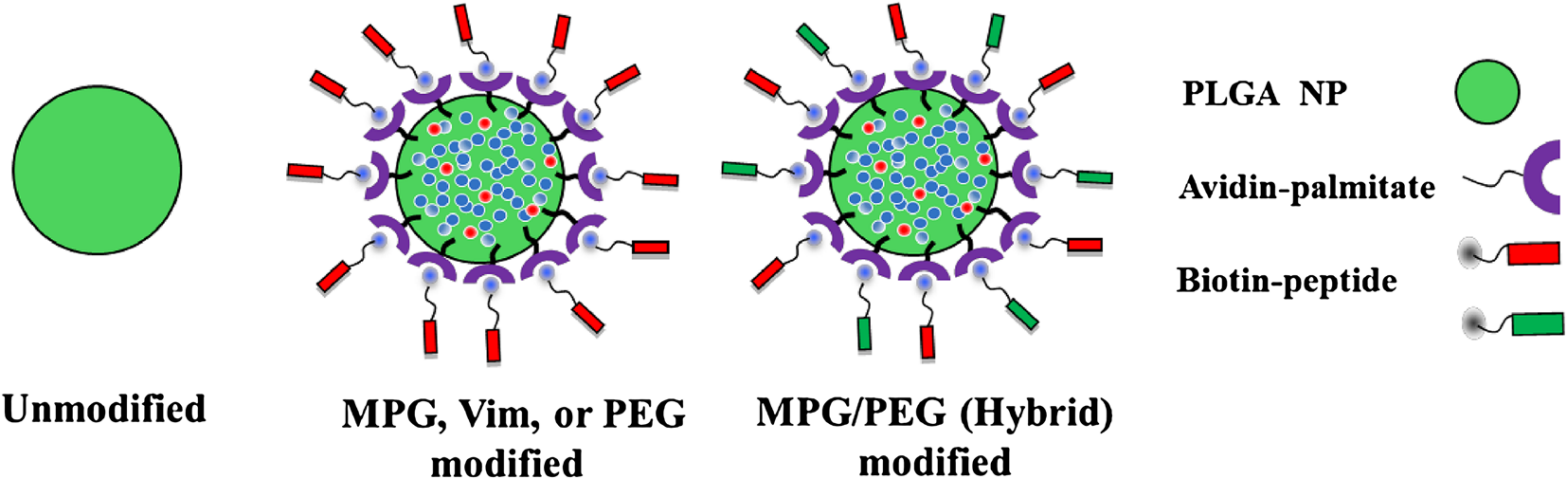
Schematic illustrating NP formulations used in this study

The NP surface was first modified with avidin-palmitate, and different NP groups were created based on the addition of one of the following biotinylated ligands: MPG, MPG/PEG, PEG, or VIM, as described in the “Methods” section. Figure 1 illustrates the NPs used in this study, while Additional file 1: Figure S1 shows a representative scanning electron microscopy (SEM) image of the unmodified NPs and their size distribution. The average unhydrated NP diameter measured 167 ± 50 nm. Hydrodynamic sizes and surface charges for the different NP groups were quantified via dynamic light scattering (DLS) and zeta potential measurements, respectively (Additional file 1: Table S1).

Unmodified NPs had a hydrodynamic diameter of 267 ± 13.6 nm and a negative surface charge of −17.3 ± 0.5 mV, and the addition of avidin-palmitate increased this surface charge to −14.3 ± 0.59 mV. The surface charge was further increased with the addition of the ligands, with MPG-modified NPs having the most positive charge at −0.4 ± 0.2 mV, followed by PEG and MPG/PEG with −5.2 ± 1.3 and −5.3 ± 0.7 mV, respectively, and VIM with −11.1 ± 1.0 mV. NP hydrodynamic sizes ranged from 232 to 277 nm for both unmodified and surface-modified groups, with no statistically significant difference between the groups.

### Uptake of PLGA-modified nanoparticles in cervical cancer cell monolayers

**Fig. 2:**
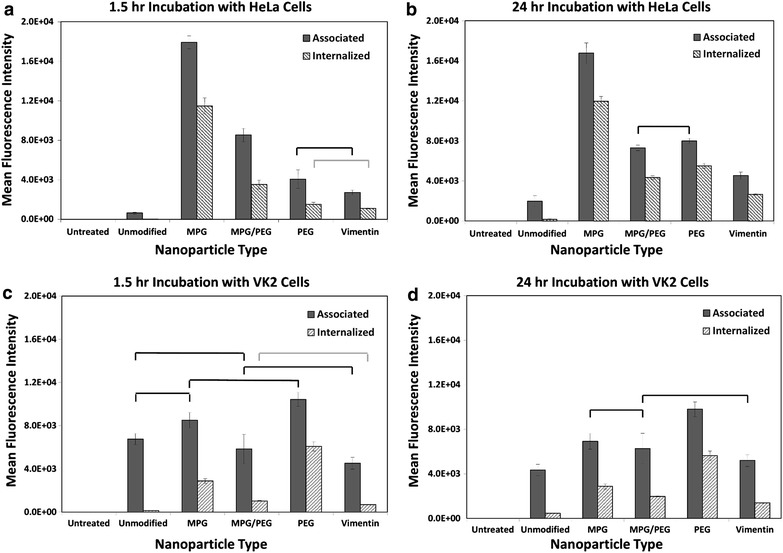
Cellular association and internalization of the various NP formulations are presented for HeLa (*top*) and VK2 (*bottom*) cells, shown at 1.5 h (*left*) and 24 h (*right*). Statistically similar results are shown linked with an *overbar*

Cellular association (binding plus internalization) and internalization in 2D cell culture monolayers were quantitatively assessed using Fluorescence Activated Cell Sorting (FACS) with cervical tumor epithelial cells (HeLa) and normal vaginal epithelial cells (VK2) (Fig. 2). Both HeLa and VK2 cells were incubated with the same concentration of NPs (0.05 mg/mL) for 1.5 or 24 h, regardless of modification. For HeLa cells, all of the modified NP groups showed both greater total association as well as internalization relative to unmodified NPs at both time points. At 1.5 h, MPG, MPG/PEG, PEG, and VIM NPs were internalized at 607×, 184×, 79×, and 57× that of unmodified NPs, respectively (Fig. 2a). Comparing the formulations to each other, the MPG NPs showed 3.3×, 7.6×, and 10.5× greater internalization than the MPG/PEG, PEG, and VIM NPs, respectively. After 24 h of incubation, MPG, MPG/PEG, PEG, and VIM NPs were internalized at 66×, 24×, 30×, and 15× that of unmodified NPs, respectively (Fig. 2b). The MPG NPs were internalized at 2.7×, 2.2×, and 4.5× that of MPG/PEG, PEG, and VIM NPs, respectively. Since the internalization for the MPG NPs was essentially the same at both time points, these data highlight the increase in internalization of the other formulations after longer exposure times.

### Uptake of PLGA-modified nanoparticles in vaginal epithelial cell monolayers

In contrast, NP association with VK2 cells at 1.5 h was not as distinct between the various modified formulations (Fig. 2c), whereas PEG NPs internalized at 2.1×, 5.9×, and 8.8× compared to MPG, MPG/PEG and VIM NPs, respectively. Comparing to unmodified NPs, the MPG, MPG/PEG, PEG, and VIM NPs internalized respectively at 20×, 7×, 43×, and 5×. After 24 h however, a clearer separation in association was apparent and PEG NPs were internalized at 1.9×, 2.8×, and 4.0× compared to MPG, MPG/PEG, and VIM NPs, respectively (Fig. 2d).

The internalization remained invariant when comparing MPG and PEG between the two time points, while the internalization of MPG/PEG and VIM essentially doubled. When compared to unmodified NPs after 24 h incubation, the MPG, MPG/PEG, PEG, and VIM internalized at 6.3×, 4.3×, 12.4×, and 3.0× that of unmodified NPs.

### Comparison of nanoparticle uptake between tumorigenic and normal cell monolayers

**Fig. 3:**
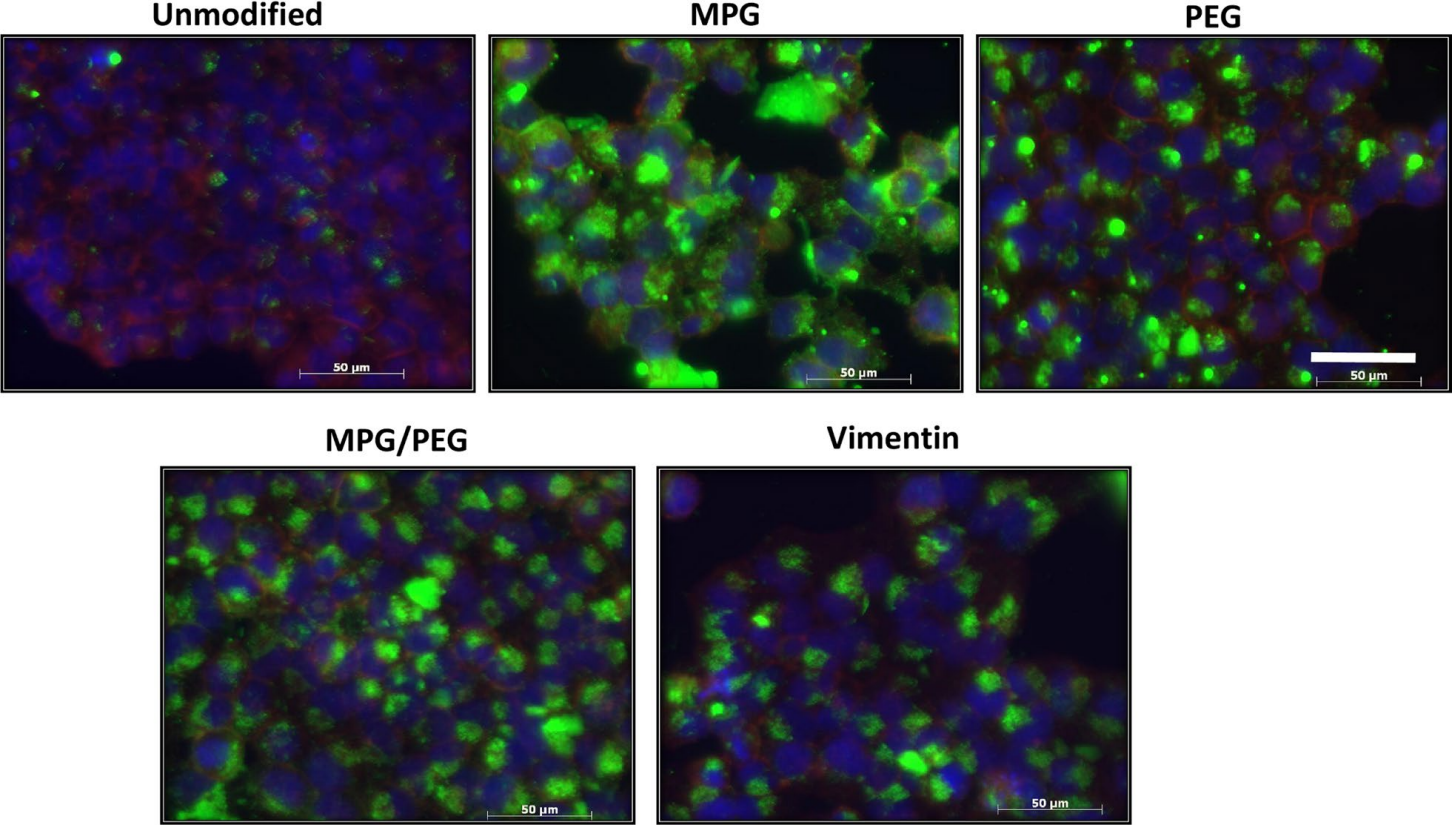
Total NP association (binding and internalization) in monolayers of HeLa cells after 24 h incubation. Nuclei are *blue* (Hoechst), actin cytoskeletons are *red* (Texas red phalloidin), and NPs are *green* (Coumarin 6). *Bar* 50 μm

**Fig. 4:**
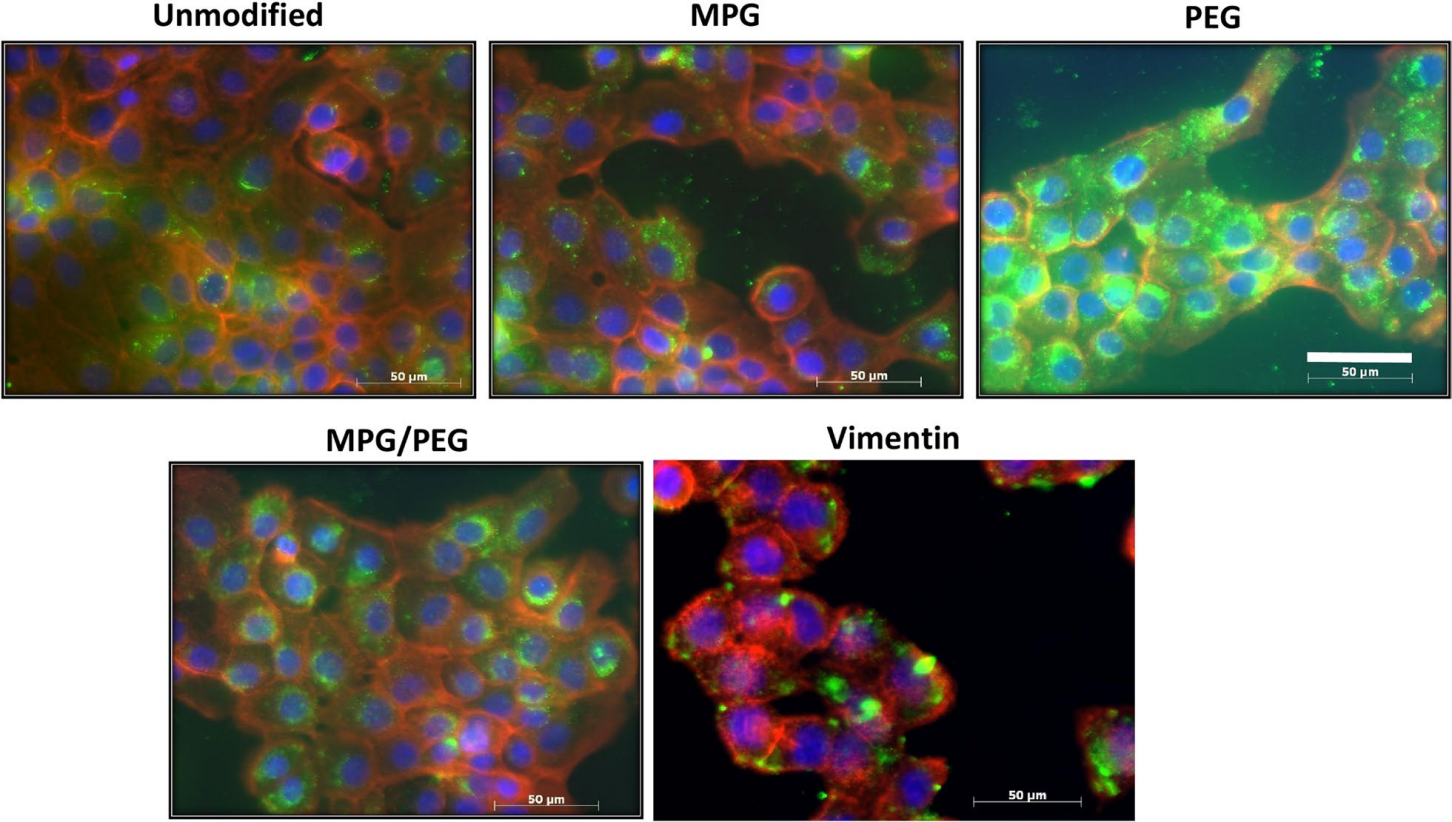
Total NP association (binding and internalization) in monolayers of VK2 cells after 24 h incubation. Nuclei are *blue* (Hoechst), actin cytoskeletons are *red* (Texas red phalloidin), and NPs are *green* (Coumarin 6). *Bar* 50 μm

Of note after 24 h, the unmodified NPs readily associate with the VK2 cells, in contrast to HeLa cells; however, unmodified NPs demonstrate low levels of internalization in both cell lines. In contrast, surface-modified NP uptake was significantly enhanced in both the HeLa and VK2 cell lines. MPG NPs were internalized the most in HeLa cells; whereas PEG NPs were internalized most highly in VK2 cells. While both surface-modified groups (MPG and PEG) demonstrated strong internalization in HeLa and VK2 cells respectively, MPG was internalized in HeLa cells 2× that of the PEG NPs by the VK2 cells. When comparing between the earlier and later time points, it is apparent that the PEG NPs showed the highest increase in both association and internalization by the longer exposure with the tumorigenic HeLa but not with the normal VK2 cells. Cellular association and internalization were qualitatively assessed via inverted epifluorescence microscopy for both HeLa and VK2 cells at 1.5 h (Additional file 1: Figures S2, S3) and 24 h incubation times (Figs. 3, 4). Although the images for the most part reflect the flow cytometry results in Fig. 2, they are representative samples providing a limited assessment.

### Uptake of PLGA-modified nanoparticles in hypo-vascularized cancer lesions

**Fig. 5:**
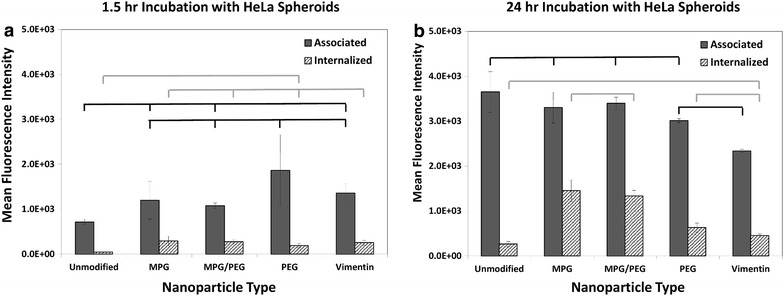
Cellular association and internalization of the various NP formulations in HeLa cell tumor spheroids. Results are shown at (**a**) 1.5 and (**b**) 24 h. Statistically similar results are shown linked with an *overbar*

Cellular association and internalization in 3D cell culture (HeLa cervical tumor spheroids), incubated with the same concentration of NPs (0.05 mg/mL) as the monolayers, were quantitatively assessed at 1.5 and 24 h via flow cytometry (Fig. 5). At 1.5 h, as a group, all of the modified NPs had higher association than the unmodified NPs, with statistically similar association when compared to each other. The surface-modified NPs also evinced a statistically similar internalization when compared to each other, but in this case, as a group the MPG, MPG/PEG, and VIM NPs showed higher internalization than the unmodified and PEG NPs (Fig. 5a). In contrast, after 24 h, the unmodified and surface-modified NPs (except for VIM, which was lower) showed statistically similar association (Fig. 5b), while MPG and MPG/PEG showed 2× and 3× internalization relative to PEG and VIM NPs, respectively. Interestingly, unlike the results obtained with the monolayer, in the 3D cell culture the association of all NPs increased significantly at 24 h compared to the 1.5 h time point: unmodified by 4.8×, MPG by 2.6×, MPG/PEG by 3.0×, PEG by 1.6×, and VIM by 1.6×. Internalization also increased at the later time point, with MPG and MPG/PEG NPs being internalized the most (at 3.6× and 3.8× compared to 1.5 h, respectively), while VIM or unmodified versions were internalized the least.

### Comparison of nanoparticle uptake between 2D and 3D cell cultures

**Fig. 6:**
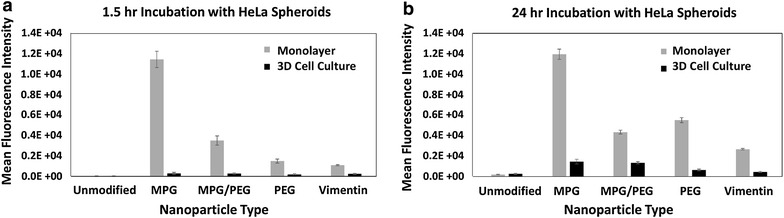
Comparison of NP cellular internalization between HeLa monolayer and spheroid cell cultures. Results are shown at (**a**) 1.5 and (**b**) 24 h

In general, all of the values measured from 3D cell cultures were lower than those from the 2D cell cultures, highlighting the effect of the diffusive transport barrier in hypo-vascularized tissue. Figure 6 summarizes the NP cellular internalization as a function of surface modification, cell culture type, and treatment duration, highlighting the effect of diffusive transport on NP uptake between the monolayer and tumor environments. It is expected that monolayer culture represents optimal conditions in terms of diffusive transport, and therefore association and internalization would be lower in the 3D tumor environment.

### Transport of PLGA-modified nanoparticles in hypo-vascularized cancer lesions

**Fig. 7:**
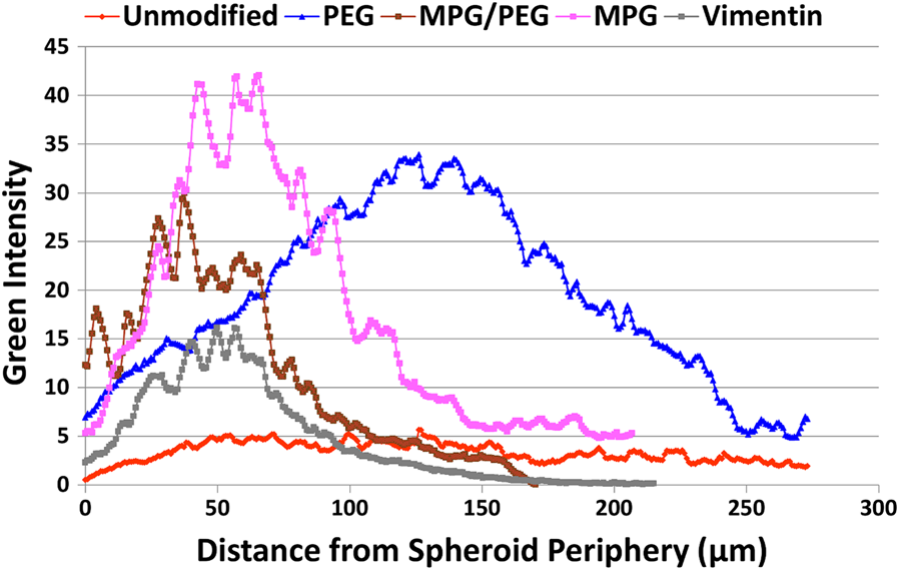
NP penetration into 3D cell culture (HeLa spheroids) after 1.5 h incubation

The NP diffusion profiles after 1.5 h through the spheroid tissue are presented in Fig. 7, showing that MPG and PEG-modified NPs exhibited the greatest fluorescence intensity averaged over 200 × 80 μm areas, followed by MPG/PEG and VIM NPs. In particular, MPG NPs were heavily detected in the periphery (within 100 μm of the edge) of the spheroids, viz., 2×, 1.4×, and 2.7× that of PEG, MPG/PEG, and VIM NPs, respectively, while PEG NPs penetrated 2× farther (250 μm) into the spheroid than the MPG NPs. In comparison, unmodified NPs exhibited the lowest detection and penetration into the tumor spheroids. Representative images for each NP type are shown in Additional file 1: Figure S4.

## Discussion

Building upon our previous work using avidin-palmitate PLGA NPs with modified surface chemistry [31], here we study the effects of contrasting and hybrid surface modifications on NP transport and cellular internalization in cervical tumor cells. Previously, avidin-palmitate NPs conjugated with the cell-penetrating peptide MPG were shown to have rapid internalization in monolayer cervical carcinoma (HeLa) cells, with a significant improvement over unmodified NPs. Here, we expand the suite of NP surface modifications to include PEG, VIM, and a hybrid modification of MPG and PEG. These formulations were evaluated for their internalization over short and extended time periods (1.5 and 24 h, respectively) in monolayer cell culture, representing optimal transport conditions, and in tumor spheroid cultures, representing hypo-vascularized tumor lesions with diffusive transport limitations. Additionally, we extend our analysis beyond cancerous cells to include normal vaginal cells (VK2) to assess potential differences in the efficacy of different surface modifications when targeting tumor versus non-tumor cells present in the female reproductive tract.

The NP formulations were characterized using standard methods to evaluate size, hydrodynamic size, and zeta potential (Additional file 1: Figure S1; Table S1). Unmodified NPs exhibited the most negative surface charge, while surface modification increased the surface potential to more positive values. MPG NPs exhibited the greatest increase in surface potential, as MPG is an amphipathic peptide that contains a cationic sequence known to enhance cell surface interaction. All NP surface modifications yielded an improvement in cellular association and internalization over unmodified NPs under optimal transport conditions in HeLa monolayer cell culture (Fig. 2a, b). MPG-modified NPs had the highest cellular association and internalization at both 1.5 and 24 h, indicating their potential to efficaciously promote NP uptake. The hybrid MPG/PEG NPs showed significantly enhanced uptake at 1.5 h, greater than both PEG and VIM-modified NPs. However, PEG significantly increased cellular internalization after a longer exposure time and surpassed the hybrid MPG/PEG NPs. The MPG, VIM, and hybrid NPs showed modest improvement in internalization after longer treatment duration. These results are in agreement with our previous work that MPG-modified NPs have a rapid mechanism of uptake in HeLa cells [31] while PEG-modified NPs take longer to internalize. This is consistent with PEG functionality, which is designed to prolong half-life in vivo.

In contrast, in normal vaginal cells (VK2), PEG-modified NPs exhibited similar high uptake and internalization at 1.5 and 24 h (Fig. 2c, d), while MPG and MPG/PEG NPs showed only slight internalization. In fact, the MPG-modified NPs demonstrated greater than threefold improvement in internalization by cancerous relative to normal cells. These results suggest that the association and uptake of these NPs in normal cells may be mediated by different molecular mechanisms or cell surface moieties than in cancerous cells, which may have significant implications in more specific targeting of cancer lesions. The results further suggest that while VIM NPs are more efficacious than unmodified NPs, VIM most likely does not serve a specific targeting purpose for these cells. Enhancement is most likely due to the slightly more positive surface potential compared to that of the unmodified NPs. The high internalization of PEG-coated NPs in the VK2 cells, and also after 24 h in HeLa cells, indicates that these NPs might provide a means to enhance transport and concentration of therapeutics into normal vaginal tissue, while also offering the potential for enhanced uptake in cancerous cells.

Following the assessment of cellular internalization in optimally diffusive (monolayer) conditions, a similar evaluation was performed in 3D cell culture of HeLa cells to assess the transport and uptake of these NPs in hypo-vascularized tumor tissue. After the short incubation of 1.5 h, all surface-modified NPs performed similarly (Fig. 5), demonstrating slightly increased internalization relative to the unmodified NPs. After 24 h, the MPG and MPG/PEG NPs exhibited the most internalization relative to the other NP groups. Unlike the monolayer cultures, none of the groups saturated the spheroids at 1.5 h, and instead elicited a more gradual, yet significant increase in internalization with longer exposure time. These results highlight the diffusive transport limitation through the 3D tissue culture as a time-dependent process, and enable a more detailed assessment of NP performance. The transport limitation in 3D tissue is well known (e.g., [52–56]), and derives from the extra-cellular matrix as well as cell–matrix and cell–cell interactions (such as E-cadherin) impeding diffusive flow [57, 58]. Whereas in monolayer HeLa and VK2 cell cultures the MPG and PEG NPs were internalized the most compared to the other groups, in the 3D cell culture the MPG and MPG/PEG NPs showed highest internalization. The diffusive transport limitation is further emphasized in Fig. 6, which compares the 2D and 3D internalization of the NPs in monolayer versus spheroid cultures.

As shown in Fig. 7, PEG NPs have the greatest depth of penetration into the spheroid tissue, with maximal concentration reached at 125 μm. In contrast, the MPG NPs achieved the highest concentration along the tumor periphery (maximally at 65 μm), but were unable to fully penetrate it. As shown in the monolayer, MPG NPs have the highest rate of cellular internalization, which is critical to the success of some biological therapeutics that cannot transverse cellular membranes alone, such as oligonucleotides, but this increased internalization may limit the depth of penetration. In contrast, the PEG modification, being very hydrophilic and less positively charged, enables NPs to more easily navigate the extracellular space and penetrate deeper into the spheroid tissue at the expense of internalization.

In living subjects, circulating NPs can preferentially lodge in tumor tissue by extravasating from fenestrated capillaries [59] as a result of the enhanced permeability and retention effect (EPR). Nevertheless, it has been shown that NP diffusive transport only extends about 30–50 μm away from the vessels [60, 61]. Consequently, therapeutics released from these NPs may not attain cytotoxic concentrations and also fail to affect quiescent (non-cycling) cells in regions distal to the vasculature [62]. Previously, we evaluated drug and NP transport in 2D and 3D cell culture, showing that nanoparticles layered with combinations of hexadecanethiol, phosphatidylcholine and high-density lipoprotein diffused deeper than PEGylated NPs into 3D cell culture representing hypo-vascularized tissue [63]. The NPs were uptaken in solid tumor tissue in vivo [64], and elicited distinctive drug release kinetics when loaded with paclitaxel or cisplatin [65]. Cytotoxicity experiments with free drug showed a substantial differential between 2D and 3D cell cultures, highlighting the increased resistance due to diffusive transport [34], although the drug-loaded layered NPs were substantially more efficacious in 3D cell culture than free-drug. While these findings are consistent with previous work showing decreased efficacy in 3D cell culture compared to monolayer [32, 60], they offer hope that modification of NPs to enhance their uptake and transport in cancerous tissue can help overcome the limitations of diffusive transport.

## Conclusions

The results of this study suggest the potential usefulness of combination treatment employing a blend of different NP formulations to achieve internalization of therapeutics in cancerous tissue, e.g., in which PEG NPs are used for deep penetration into tissue distal from vasculature while MPG particles target proliferative regions close to blood vessels. Pathological examination of biopsy tissue could help to inform this approach. Interestingly, while the hybrid MPG/PEG NPs had similarly high uptake as MPG after 24 h in 3D cell culture, this group did not benefit from the combination of the cell penetrating and stealth attributes, and actually had a lower uptake and penetration depth than either MPG or PEG alone. In this case, internalization and diffusion may be competitive and thus inhibit overall NP uptake and transport. These results highlight the well-known need for a proper balance of NP properties to achieve both adequate internalization and diffusion, and emphasize that NP delivery assessment would benefit not only from consideration of different time points but also from evaluation of diffusive transport.

Future studies could potentially assess in further detail the time-dependent uptake of each NP group in monolayer as well as in 3D cell culture. The delivery of therapeutic agents (drug, siRNA, proteins) could be studied to assess efficacy. Evaluation in vivo would help establish NP distribution and clearance, as well as systemic toxicity. Additionally these studies would benefit from experimental as well as computational analysis [34, 42, 44, 66–69] to elucidate the complex interactions between NP parameters (surface modifications and transport), cell types (tumorigenic vs. normal), and the tumor microenvironment (cellular proliferation and death as a function of heterogeneous oxygen, nutrients, NP, and drug concentrations).

## Methods

### Synthesis of avidin-palmitate conjugates

Avidin-palmitate was conjugated to NP surfaces as previously described [31, 70, 71] for subsequent reaction with biotinylated ligands: MPG, PEG, VIM, and an equimolar combination of MPG/PEG. Briefly, 40 mg of avidin was dissolved in 4.8 mL of 2 % sodium deoxycholate (NaDC) in phosphate buffered saline (PBS) warmed to 37 °C. Palmitic acid-N- hydroxysuccinimide ester (PA-NHS, Sigma) was dissolved in 2 % NaDC at 1 mg/mL and sonicated until well-mixed. 3.2 mL of the 1 mg/mL PA-NHS solution was added dropwise to the reaction vial, and reacted overnight at 37 °C. The following day, the reaction was dialyzed overnight in 1200 mL of 0.15 % NaDC in PBS heated to 37 °C. Free PA-NHS was removed using 3500 molecular weight cut off (MWCO) dialysis tubing, and the dialysis cassette contents were subsequently transferred to a vial and stored at 4 °C.

### Nanoparticle synthesis

We synthesized and characterized PLGA NPs encapsulating a fluorescent dye, Coumarin 6 (C6), to evaluate tumor penetration and distribution via fluorescence microscopy. From earlier studies [72–75] as well as our previous experiments, we have observed that negligible quantities (~1 %) of C6 are released from NPs. This is attributed to the hydrophobic nature of C6 encapsulated within hydrophobic NPs. Therefore, C6 detected in cells reflects NP distribution in or on the cells, not C6 release and distribution. C6 NPs were synthesized as previously described using an oil-in-water (o/w) single emulsion technique [31, 71]. Briefly, C6 was encapsulated into 100-200 mg PLGA carboxyl-terminated polymer (0.55–0.75 dL/g, LACTEL^®^). C6 was dissolved in methylene chloride (DCM) overnight at a concentration of 15 μg C6 per mg of PLGA. The following day, the solution was added dropwise to a 5 % polyvinyl alcohol (PVA) solution of equal volume, vortexed and sonicated. The resulting NPs were hardened during solvent evaporation for 3 h. For unmodified NPs, the NPs were washed after hardening, and centrifuged 3 times at 4 °C in deionized water (diH_2_O) to remove residual solvent. For avidin-palmitate surface-modified NPs, a similar protocol was followed [31, 71, 73]. NP formulations were synthesized by adding (1 mg/mL) avidin-palmitate to the 5 % PVA solution. NPs were collected after the first wash and incubated for 30 min with biotinylated ligands at a molar ratio of 3:1 ligand:avidin in PBS. After conjugation, the NPs were washed two more times with diH_2_O by centrifugation and subsequent washing. All NPs were frozen, lyophilized, and stored at −20 °C until use.

### Cell culture

VK2/E6E7 vaginal epithelial (VK2) and human cervical carcinoma (HeLa) immortalized cell lines were kindly provided by Dr. Kenneth Palmer’s lab (University of Louisville). These cell lines were obtained and authenticated through ATCC. We selected the VK2/E6E7 cell line, since for intravaginal delivery these would be the first “normal” cells to encounter NP treatment. HeLa cells provide the ability to assess NP behavior against a cervical cancer cell line in vitro. VK2/E6E7 vaginal epithelial cells (VK2) were maintained in Defined Kerotinocyte-Serum Free medium (SFM) supplemented with Defined Keratinocyte-SFM Growth Supplement. HeLa cells were maintained in minimal essential media (MEM) supplemented with 10 % fetal bovine serum and 1 % penicillin–streptomycin in standard culture conditions. Cell media was checked and changed daily.

### Tumor spheroid formation

HeLa cells were used for tumor spheroid formation. Cells were grown to 80 % confluence before harvesting. Twenty-four well tissue culture plates (Corning) were coated with a 1 % (w/v) agarose gel 24 h before spheroid formation to prevent cell adherence. For formation, 100,000 cells were placed in each well and lightly shaken (100 rpm) for 15 min on a reciprocating shaker. After 7–14 days of incubation, spheroid formation occurred by self-aggregation to sizes ranging from 500 to 1000 μm.

### Monolayer (2D) cellular uptake and microscopy

2D cellular imaging of 1.5 and 24 h NP uptake and penetration in HeLa and VK2 cells was performed via inverted epifluorescence microscopy [31]. VK2 and HeLa cells were seeded 24 h prior to NP administration in LabTek 8-well chamber slides at a density of 50,000 and 40,000 cells per well for 1.5 and 24 h uptake, respectively. For NP administration, NPs were massed out and dissolved to reach a final stock concentration of 0.6 mg/mL in PBS++ (containing CaCl_2_ and MgCl_2_) to aid cell adherence. One hundred microliters of fresh media were added to the cells, and NPs were then added to obtain a final NP concentration of 200 μg/mL.

After either 1.5 or 24 h incubation, cells were washed five times in 0.5 mL of 1X PBS to remove any unbound or non-internalized NPs. Cells were then fixed with 0.3 mL of 4 % paraformaldehyde and incubated for 10 min at room temperature (RT). Cells were subsequently washed twice with 0.5 mL of PBS and permeabilized with 0.3 mL of 0.1 % Triton X-100 in 1 % bovine serum albumin (BSA) PBS++ for 10 min at RT. After permeabilization, cells were incubated with 0.3 mL of 1:40 Texas Red Phalloidin in 1 % BSA PBS++ for 20 min at RT for cytoskeleton staining and were subsequently washed twice with 0.5 mL PBS. Cells were then incubated with 0.3 mL of 4 μg/mL Hoechst in 1 % BSA PBS++ for 10 min at 37 °C for nuclear staining. Finally, cells were washed twice in PBS and once in diH_2_O, then mounted in Vectashield non-hardening mounting medium (Vector Laboratories, VWR) and kept at 4 °C until imaged [31].

Inverted epifluorescence microscopy was utilized to assess cellular uptake of NPs in 2D monolayers. Briefly, cells were prepared as described above and imaged in 8-well LabTek chamber slides using the following filter settings: 4′,6-diamidino-2-phenylindole (DAPI) to visualize Hoechst, green fluorescent protein (GFP) for C6, and the Texas Red channel to evaluate NP uptake in 2D. Exposure times for DAPI, GFP, and Texas Red were kept consistent throughout experiments and were as follows: DAPI at 45 ms (excitation/emissions: 358/461 nm); GFP at 60 ms (593/615 nm); and Texas Red at 180 ms (488/515 nm).

### Spheroid (3D) cellular uptake and microscopy

To assess the differences in NP uptake and distribution through hypo-vascularized tumor tissue, HeLa spheroids were incubated with 0.01 mg/mL of NPs and visualized using confocal microscopy. Diffusion profiles through the spheroids for each NP formulation were evaluated by quantifying the fluorescence intensity in z-stack images as a function of distance from the spheroid periphery. After NP administration, tumor spheroids were transferred to LabTek 8-well chamber slides for fluorescent staining and were washed five times with 0.2 mL 1X PBS. Tumor spheroids were subsequently incubated for 10 min at RT with 0.2 mL 4 % paraformaldehyde for spheroid fixation. Following fixation, spheroids were washed twice with 0.2 mL of PBS, followed by incubation with 0.2 mL of 1:40 Texas Red Phalloidin in 1 % BSA PBS++ for 20 min at RT for cytoskeleton staining. Spheroids were then washed twice with 0.2 mL PBS and incubated with 0.2 mL of 4 μg/mL Hoechst in 1 % BSA PBS++ for 10 min at 37 °C for nuclear staining. Finally, spheroids were washed twice in 0.2 mL PBS and once in 0.2 mL diH_2_O. Spheroids were then mounted with Vectashield non-hardening mounting medium. 3D uptake and distribution of NPs through tumor spheroids was assessed using confocal microscopy. Images were processed using ImageJ by taking representative samples from tumor cross-sections (≥90 averaged profiles for each sample).

### Flow cytometry analysis

Cells were plated in 6-well plates (Corning) at a density of 200,000 cells per well. Both VK2 and HeLa cell lines were incubated with NP suspensions at 0.05 mg/mL for either 1.5 or 24 h in a 37 °C humidified chamber in the appropriate medium [31]. An unmodified NP control group was used to compare uptake relative to surface-modified NPs, as our previous work demonstrated minimal difference between unmodified and avidin-modified NP association and uptake [31]. After incubation, the cells were washed five times with PBS++. Next, the cells were dissociated with enzyme-free cell dissociation buffer (ThermoFisher). The dissociated cells were moved to FACS tubes, centrifuged, and resuspended in a FACS buffer solution containing 1 % BSA and 0.1 % sodium azide. From each sample, half the cells were moved to separate FACS tubes and kept on ice until analyzed (total associated samples). The remaining cells were exposed to 0.4 % trypan blue for 5 min to quench extracellular fluorescence, washed twice in FACS buffer, and kept on ice until analyzed (internalized samples) [31]. All FACS tubes from both groups were analyzed using a BD LSRFortessa Flow Cytometer (BD Biosciences). Data were analyzed using FlowJo software (FlowJo Enterprise), and a minimum of 10,000 cells were analyzed per sample. For flow cytometry analysis of tumor spheroids, a similar protocol was followed using spheroids after 7 days of growth. Instead of enzyme-free cell dissociation buffer, 0.25 % Trypsin–EDTA was used to fully disaggregate the spheroids before centrifugation and resuspension in FACS buffer solution.

### Statistical analysis

Experiments were conducted each with a minimum sample size of n = 3. Data were analyzed by applying Tukey’s test with significance p < 0.05. Unless otherwise noted, all figure error bars represent the standard deviation of the measurements. To enable clear interpretation, statistically similar results are shown linked with an overbar in the figures of the “Results” section.

## Additional file

10.1186/s12951-016-0185-x NP characterization. Representative scanning electron microscopy image of **a** unmodified NPs and **b** the corresponding size distribution. **Figure S2.** Total NP association (binding and internalization) in monolayers of HeLa cells after 1.5 hr incubation. Nuclei are blue (Hoechst), actin cytoskeletons are red (Texas red phalloidin), and NPs are green (Coumarin 6). Bar = 50 μm. **Figure S3.** Total NP association (binding and internalization) in monolayers of VK2 cells after 1.5hr incubation. Nuclei are blue (Hoechst), actin cytoskeleton are red (Texas red phalloidin), and NPs are green (Coumarin 6). Bar = 50 μm. **Figure S4.** Total NP association (binding and internalization) in spheroids of HeLa cells after 1.5 hr incubation. Nuclei are blue (Hoechst) and NPs are green (Coumarin 6). Spheroid periphery is highlighted by the brighter blue color, with the exterior showing in black. Magnification: 15x. **Table S1.** Hydrodynamic sizes and zeta potentials measured for the various NP formulations.

## Abbreviations

2D: two-dimensional
3D: three-dimensional
BSA: bovine serum albumin
C6: Coumarin 6
DAPI: 4′,6-diamidino-2-phenylindole
diH_2_O: deionized water
FACS: Fluorescence Activated Cell Sorting
GFP: green fluorescent protein
MWCO: molecular weight cut off
NaDC: sodium deoxycholate
NP: nanoparticle
PA-NHS: palmitic acid-N-hydroxysuccinimide ester
PBS: phosphate buffered saline
PEG: polyethylene glycol
PLGA: poly(lactic-co-glycolic) acid
PVA: 5 % polyvinyl alcohol
RT: room temperature
SEM: scanning electron microscopy
VIM: Vimentin tubulin binding site peptide
