# Supplementary material for: Enhanced uptake and transport of PLGA-modified nanoparticles in cervical cancer
Source: J Nanobiotechnology. 2016 Apr 22;14:33. doi: 10.1186/s12951-016-0185-x (PMC4840861; doi:10.1186/s12951-016-0185-x)
Supplement: Supplementary file 1 — 10.1186/s12951-016-0185-x NP characterization. Representative scanning electron microscopy image of a unmodified NPs and b the corresponding size distribution. Figure S2. Total NP association (binding and internalization) in monolayers of HeLa cells after 1.5 hr incubation. Nuclei are blue (Hoechst), actin cytoskeletons are red (Texas red phalloidin), and NPs are green (Coumarin 6). Bar = 50 μm. Figure S3. Total NP association (binding and internalization) in monolayers of VK2 cells after 1.5hr incubation. Nuclei are blue (Hoechst), actin cytoskeleton are red (Texas red phalloidin), and NPs are green (Coumarin 6). Bar = 50 μm. Figure S4. Total NP association (binding and internalization) in spheroids of HeLa cells after 1.5 hr incubation. Nuclei are blue (Hoechst) and NPs are green (Coumarin 6). Spheroid periphery is highlighted by the brighter blue color, with the exterior showing in black. Magnification: 15x. Table S1. Hydrodynamic sizes and zeta potentials measured for the various NP formulations. [file 12951_2016_185_MOESM1_ESM.pdf]

Supplementary Material for:

Enhanced Uptake and Transport of PLGA-modified Nanoparticles in Cervical Cancer

Lee B. Sims[1,+], Louis T. Curtis[1,+], Hermann B. Frieboes[1,2,3,§] Jill M. Steinbach-Rankins[1,3,4,5.§,*]

[1]Department of Bioengineering, University of Louisville, Louisville, KY, USA

[2]James Graham Brown Cancer Center, University of Louisville, Louisville, KY, USA

[3]Department of Pharmacology and Toxicology, University of Louisville, Louisville, KY, USA

[4]Department of Microbiology and Immunology, University of Louisville, Louisville, KY, USA

[5]Center for Predictive Medicine, University of Louisville, Louisville, KY, USA

[+] Equal contribution.

[§] Joint senior authorship.

*** Correspondence:** Jill M. Steinbach-Rankins, Department of Bioengineering, 505 S. Hancock, CTRB 623, University of Louisville, KY 40208, USA. Tel.: 502-852-5486; Fax: 502-852-6806; E-mail: jmstei01@louisville.edu

Contents:

Additional file Table S1

Additional file Figures S1, S2, S3, and S4

Table S1. Hydrodynamic sizes and zeta potentials measured for the various NP formulations.

**Nanoparticle Type**	**Hydrodynamic Diameter (nm)**	**Zeta Potential (mV)**
Unmodified	267.0 ± 13.6	-17.3 ± 0.5
MPG	232.6 ± 12.7	-0.4 ± 0.2
MPG/PEG	240.1 ± 49.4	-5.3 ± 0.7
PEG	239.5 ± 12.7	-5.2 ± 1.3
VIM	277.5 ± 46.8	-11.1 ± 1.0

Additional file Figures

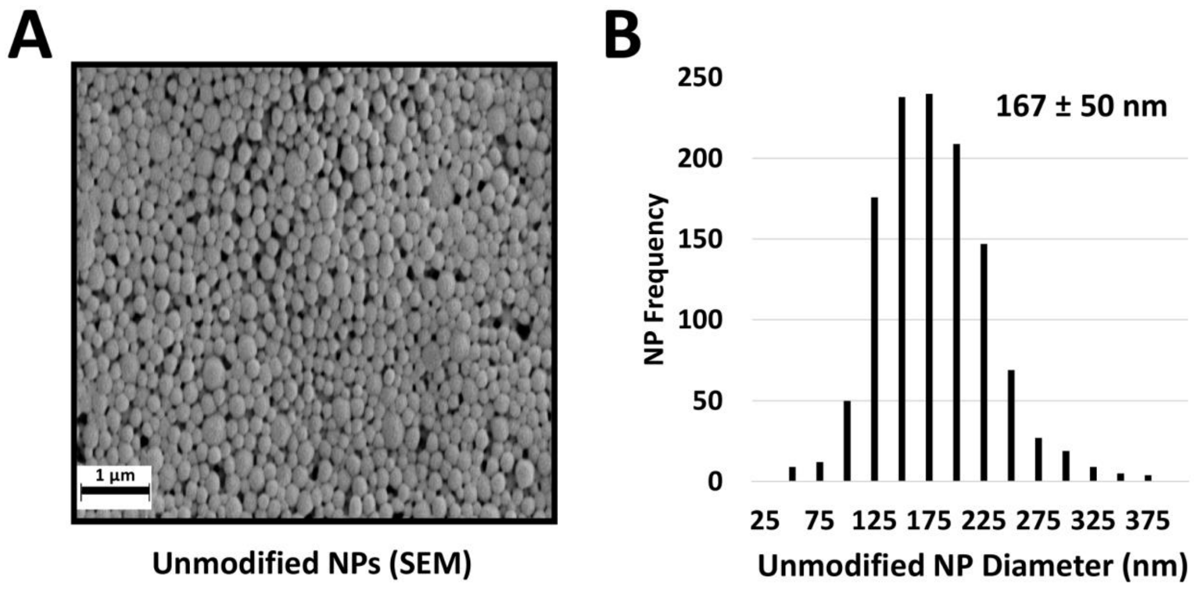

Figure S1. NP characterization. Representative scanning electron microscopy image of (A) unmodified NPs and (B) the corresponding size distribution.

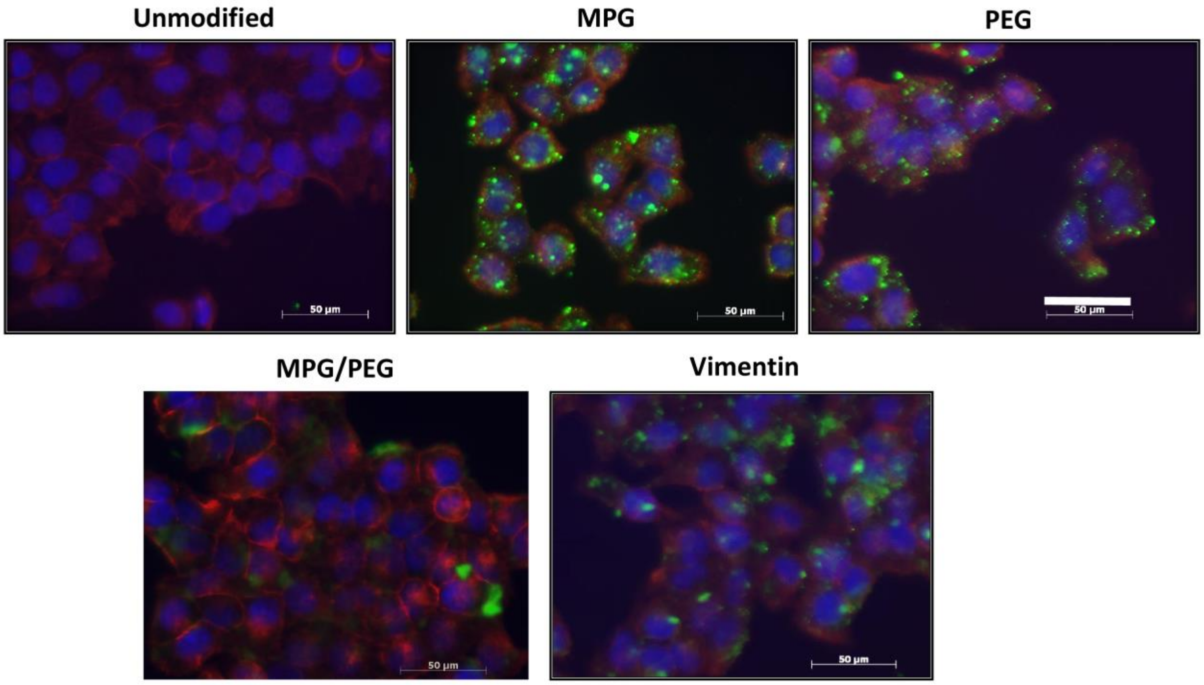

Figure S2. Total NP association (binding and internalization) in monolayers of HeLa cells after 1.5hr incubation. Nuclei are blue (Hoechst), actin cytoskeletons are red (Texas red phalloidin), and NPs are green (Coumarin 6). Bar = 50 μm.

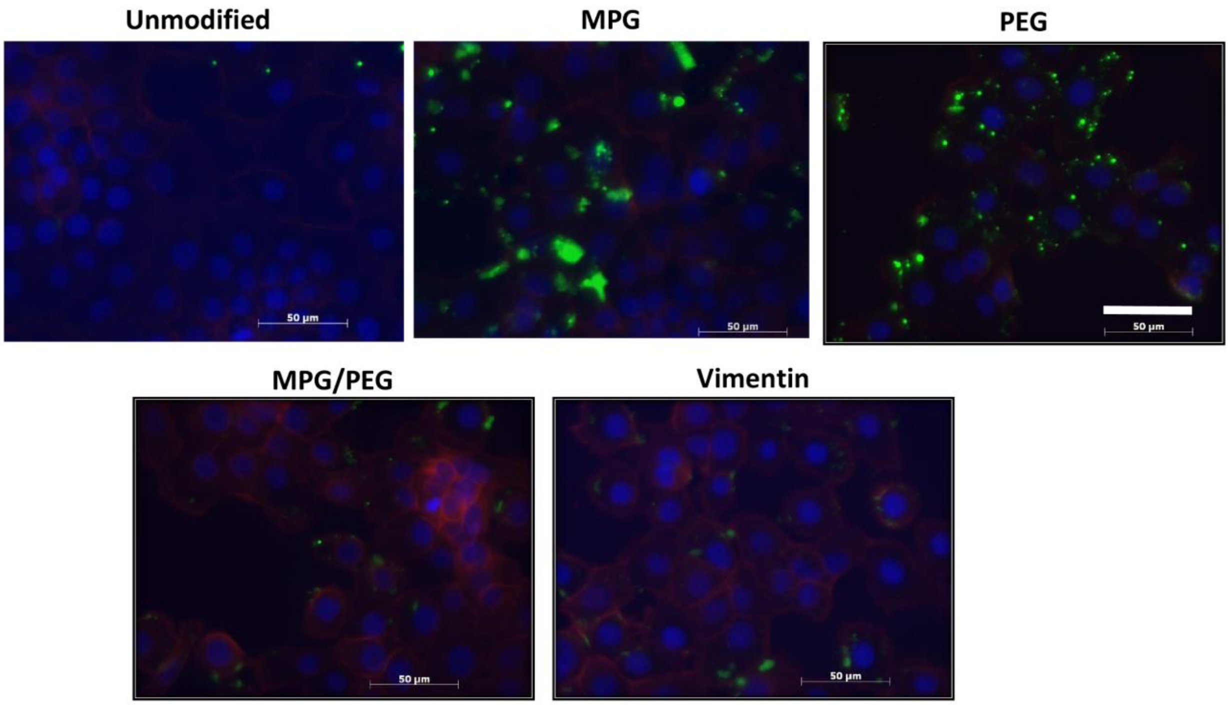

Figure S3. Total NP association (binding and internalization) in monolayers of VK2 cells after 1.5hr incubation. Nuclei are blue (Hoechst), actin cytoskeleton are red (Texas red phalloidin), and NPs are green (Coumarin 6). Bar = 50 µm.

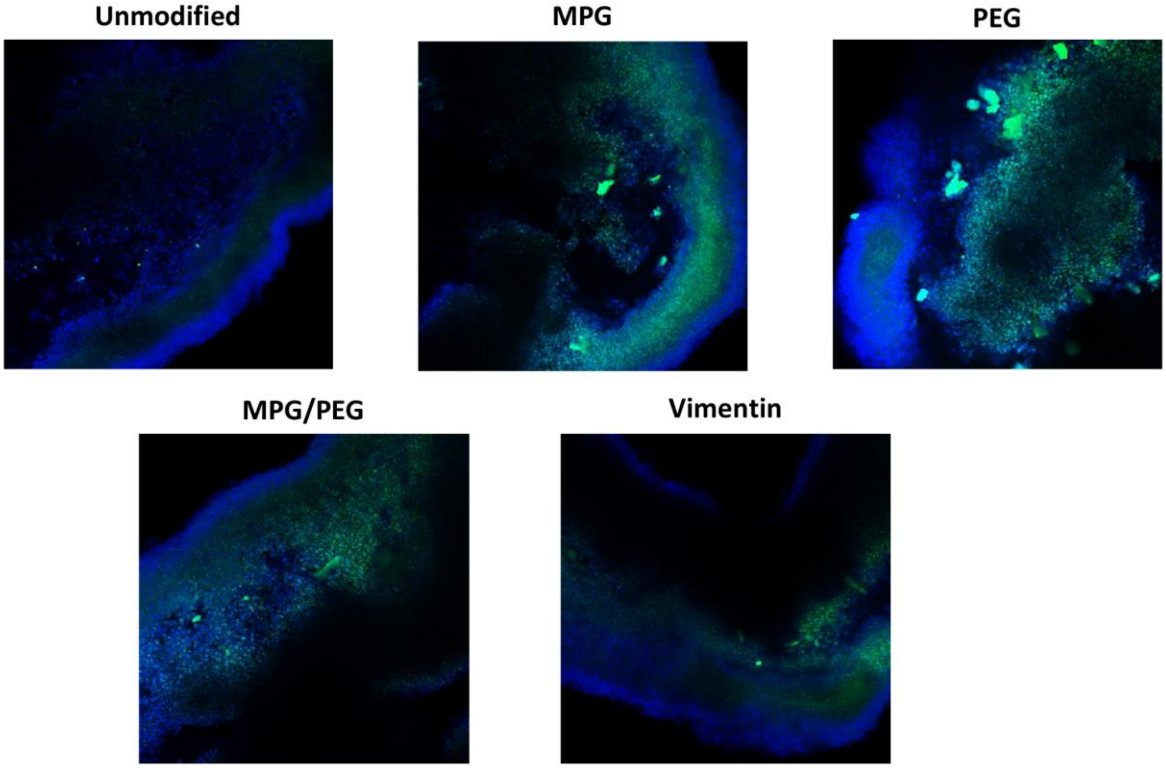

Figure S4. Total NP association (binding and internalization) in spheroids of HeLa cells after 1.5hr incubation. Nuclei are blue (Hoechst) and NPs are green (Coumarin 6). Spheroid periphery is highlighted by the brighter blue color, with the exterior showing in black. Magnification: 15x.
